# An Intelligent Fuzzy Rule-Based Personalized News Recommendation Using Social Media Mining

**DOI:** 10.1155/2020/3791541

**Published:** 2020-05-31

**Authors:** Saravanapriya Manoharan, Radha Senthilkumar

**Affiliations:** Department of Information Technology, Madras Institute of Technology, Anna University, Chennai, India

## Abstract

Recommendation of a relevant and suitable news article is an essential but a challenging task due to changes in the user interest categories over time. Moreover, the Internet technology provides abundant news articles from a huge amount of resources. Meanwhile, nowadays, many people are confronted with viral news articles through social media cost-free without considering the news sites. Therefore, mining of social media for addressing such viral news articles has become another key challenge. To overcome the above challenges, this paper proposes fuzzy logic approach for predicting users' diversified interest and its categories by analysing their implicit user profile. Depending on users' interest categories, the viral news articles and their categories were determined and analysed through mining social media feeds-Facebook and Twitter. Furthermore, fresh news articles are retrieved from news feeds incorporated with retrieved viral news articles provided as recommendation with respect to users' diversified interest. The performance of the proposed approach for predicting overall users' interest for all categories attained 84.238%, and recommendation accuracy from News feed, Facebook, and Twitter attained 100%, 90%, and 100% with respect to users' interest categories.

## 1. Introduction

With the advancement of World Wide Web (WWW), news reading style of people has rapid developments in Internet, and news reading pattern of people has slowly changed from the traditional print model to the Internet [1]. As the Internet provides access to news articles of a large variety from numerous resources, reading online news articles has become popular and daily routine for many people. Nevertheless, it is a tough task for news readers to identify desirable news articles relevant to them [2]. For mitigating this problem of information overload, news recommendation system plays a vital role for news portals. The news recommendation has certain unique challenges [2] compared to other domain recommendations such as item, movie, and music as the relevance of news articles may change rapidly with a short interval time relating to every recent event happening around the world. However, the news sites update the news articles immediately every time, but some news articles may be outdated by breaking news on the same topic multiple times persistently throughout the day requiring a constant update in the recommendation process. Moreover, individuals in news article reading are topic-sensitive; as a result, they are usually interested in several news categories that include entertainment and sports. Thus, prediction of users' interest on those categories based on their reading habits is a challenging task.

In addition to that, nowadays, people get news articles from social media rather than from news sites. The news articles that can be spread in online social media become viral and reach the people instantly due to general open discussions [3]. This discussion helps in identifying various viral news and events occurring in the entire world. People show much interest in obtaining such viral and fresh news articles. So, it becomes a need to frame a system which recommends such viral news articles from social media as well as fresh news articles from the news channel purely based on users' diversified reading habits. In this paper, we present a fuzzy logic approach to predict users' diversified interest because fuzzy logic has been extensively used in recommendation systems to handle uncertainty, impreciseness, and vagueness from the users' behaviour and make optimal prediction of users' interest [4, 5]. In this paper, Mamdani fuzzy inference system is used for prediction of users' interest for the following categories, namely, business, sports, technology, entertainment, and politics.

In many of the previous works [6–9], news articles were recommended absolutely based on the users' interest. However, they did not give attention on recommending the viral news articles obtained from social media. Few researchers focused on this issue, and they recommended news articles by harnessing social media feed—Twitter [10–13]. This work determines the viral news articles from Twitter and also additionally from Facebook. Many people come across the news articles on Facebook as a result of their friends' shares or likes. So, Facebook becomes the best channel for news article sharing [3, 14].

The major contributions of this work are summarized as follows:

1. Fuzzy logic approach to predict users' diversified interest in various news categories using the Mamdani fuzzy inference system
2. Determination and retrieval of viral news articles by mining Twitter and Facebook feeds
3. Recommendation of the fresh news articles along with viral news articles with respect to users' interest categories retrieved from social media

The rest of the paper is framed as follows.  Section 2 discusses the related works with regard to popularity-based and personalized recommendation.  Section 3 describes the proposed work and its architecture and a brief description of dataset construction and its analysis.  Section 4 deals with the experimental results of the proposed work.  Section 5 provides the conclusion and the possibilities of future work.

## 2. Literature Review

Recommendation system (RS) plays a vital role in e-commerce. It helps the people tackling the problem of information overloading [15–17]. The main objective of the RS is to filter information from several resources according to users' interests or preferences. During the past decades, noteworthy improvements have been made in recommendation. Recommendation systems have been applied in many fields that include movie (movie recommendation) [18], item (item recommendation) [19, 20], news (news recommendation) [21], and music (music recommendation) [22]. In contrast, to all the aforementioned recommendation systems, news recommendation systems generally have certain unique characteristics which are not present in other RS [2]. It helps people to get instant updation with the outside world. Moreover, it suggests news articles to the individuals with respect to their interest. In order to make it personalized, RS builds and maintains the user profile for every user that captures people's interest over time using explicit or implicit information. The explicit information is obtained in the form of ratings, like/dislike directly given by the users [23]. Similarly, the implicit information is obtained in the form of monitoring and interpreting users' browsing history [24]. Substantial research works were carried out in the news recommendation system using either explicit or implicit user profile for the prediction of users' interest and recommendation through applying different techniques. Won-Jo Lee et al. [25] have predicted users' interest from their profile using the term frequency and inverse document frequency (TF-IDF) methodology. They have designed user profiles by observing the users' Twitter activities (hashtag, tweet, and retweets). Billsus and Pazzani [6] designed an intelligent agent which predicted users' short-term and long-term interest by applying multistrategy machine learning approaches, namely, nearest neighbour and naïve Bayes classifier. Liu et al. [21] proposed Bayesian framework for the prediction of users' interest from their implicit profile. Moreover, they experimented on Google news service obtaining better recommendation results. Bai et al. [26] proposed a novel method for the prediction of users' interest from users' implicit profile. Here, the user profile was designed by monitoring users' interaction with the news sites and their search histories. Hsieh et al. [27] proposed the channel-aware latent Dirichlet allocation technique for predicting users' interest from their profile. The user profile was constructed through monitoring the user details tracked from their e-mail, Facebook, and Twitter activities. Saranya and Sudha Sadasivam [28] proposed the rough set-based collaborative filtering technique for imputing the missing news categories for each user. Piao et al. [29] suggested news recommendation architecture using Mahout and a main memory database. The news preference rate of users was calculated on the basis of time spent by users on reading specific news articles. They built the user profile from users' smart device-accessed web pages. They determined that users preferred news categories using the naive Bayes classifier. Adnan et al. [30] proposed fuzzy logic-based content-based news recommendation for a set of articles related to other articles read by the user. They constructed implicit user profile by crawling bdnews24.com site using Google analytics. However, in this proposed work, the implicit user profile has been built and monitored using a proxy agent, and users' interests were predicted accurately using fuzzy logic approach—Mamdani fuzzy inference system—for personalized recommendation.

Besides, it is essential to include trends and popular news articles through mining social media for improving the personalized recommendation system. Natarajan and Moh [31] proposed the popularity-based news recommendation system based on users' preferred location, popularity, and trends. They determined trendy news articles by analysing social media—Twitter for providing good recommendations. Jonnalagedda et al. [10] proposed the popularity-based news recommendation system using microblogging service (tweets) along with the personalized system. They ranked news articles by identifying most popular news articles through analysis of the Twitter public timeline. However, in our perception, none of the research works was focused on mining Facebook feed to determine the most popular (viral) news articles. In our proposed work, we have addressed this issue of improving popularity-based recommendation for providing better recommendation results.

## 3. Proposed Work

**Figure 1:**
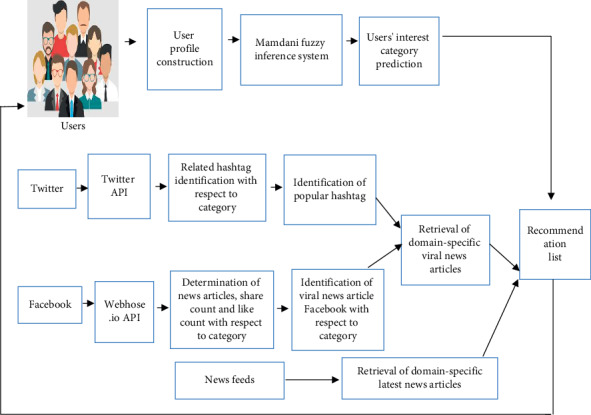
Architecture of the news recommendation framework.

This article contains three-fold contributions. The first is users' diversified interest category prediction from their implicit profile (reading history) using the Mamdani fuzzy inference system (MFIS). The second is analysis and retrieval of viral news articles by exploring social media, Twitter and Facebook feeds, for selective familiar categories. Third is recommendations of the fresh news articles from RSS feeds as well as viral news articles from social media feeds based on users' interest categories.  Figure 1 depicts the overall architecture of the news recommendation framework.

### 3.1. Data Collection and User Profile Construction

The user profiles represent users' preferences and interests and are designed to enhance the effectiveness of a personalized news recommendation system. It can be built and maintained for each individual user. In general, user profiles are of two types, namely, explicit and implicit. An explicit user profile (EUP) can be built from explicit information provided directly by users. It includes the user name, address, and specific interests in different categories of news including sports and business. Nevertheless, some people prefer not to voluntarily provide feedback or information about their interests. Inference of their interest requires tracking their browsing histories called the implicit user profile (IUP) which can be constructed through a proxy agent. The IUP contains search queries posted on the search engine, URLs visited, title and date, along with the time. In this proposed work, only IUP contents have been considered for obtaining users' interest categories.

**Figure 2:**
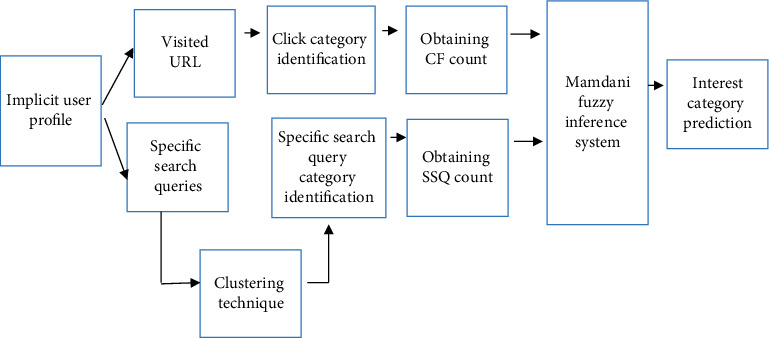
Architecture for users' interest prediction from the IUP.

Moreover, the visited URLs have been analysed, and click frequent count (CF) for specific categories is obtained using Directory.Mozilla.Org (DMOZ). Search query categories (posted on search engines) have also been analysed, and specific search query count (SSQ) is determined using a clustering technique. In order to predict users' diversified interest categories, the CF and SSQ were passed as input attributes for the Mamdani fuzzy inference system (MFIS).  Figure 2 illustrates the overall architecture for users' interest prediction from the IUP whose contents were monitored for 25 users for fifteen days continuously.

### 3.2. Methodology

**Figure 3:**
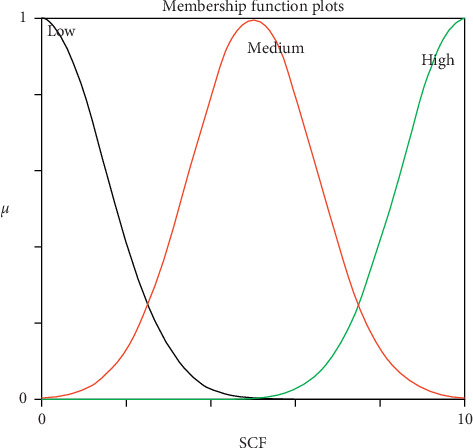
SCF input membership function.

**Figure 4:**
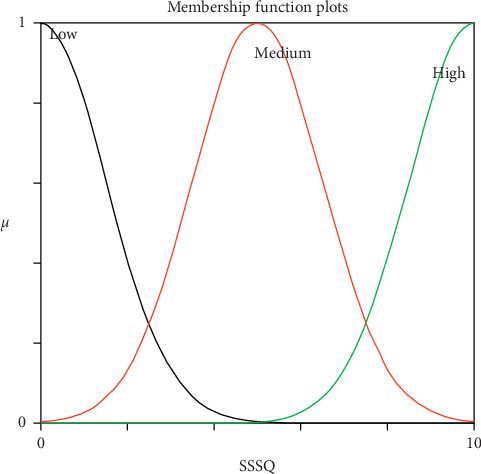
SSSQ input membership function.

**Figure 5:**
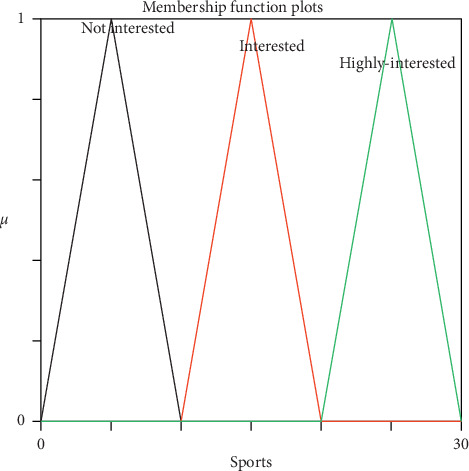
Sports output membership function.

Generally, reasoning the users' interest categories from their IUP is a challenging task because there is uncertainty associated with how to represent and infer user interest categories precisely. Mamdani fuzzy inference system (MFIS) was applied for overcoming this problem and predicting users' interest. It is a framework which handles uncertainty and imprecision, helping the people to make decisions through the degree of membership function and linguistic terms [32–34]. It mimics human interest and represents using If-Then rules. It is flexible (an easy-to-modify FIS by just adding or deleting rules) and reliable (enough to model vagueness and subjectivity in the attributes). The following section discusses the modelling of MFIS and its application to predict users' diversified interest categories. The first and foremost modelling of MFIS is fuzzification process of input attributes “CF” and “SSQ” and output attribute “Interest prediction for specific categories” which were converted from crisp values into fuzzified value using membership function [35]. The proposed MFIS model was designed with a combination of Gaussian membership function for input attributes “CF” and “SSQ” and triangular membership function for the output attribute “Interest prediction for specific category.” The Gaussian membership function has been chosen for smoothness, and triangle membership function has chosen for its monotonicity. Nevertheless, the trial and error method was utilized to get the optimum membership functions. Figures 3 and 4 represent the schematic view of the input membership function, and Figure 5 represents the output membership function for the sports category. The input membership function for sports click frequency (SCF) and sports specific search query (SSSQ) can be classified into “Low,” “Medium,” and “High” with different degrees of fuzzy sets, respectively. Similarly, the output membership function can be classified into “Not interested,” “Interested,” and “Highly-interested” with different degrees. To form rule base, modelling of MFIS is done using fuzzy “if-then” rules [36], and thus, the relationship between the input and output attributes was represented. By the knowledge of domain, experts have developed the following rules and stored in rule base.

The sample rules are as follows:

  Rule 1: if SCF is low and SSSQ is low, then sports is not interested
  Rule 2: if SCF is low and SSSQ is medium, then sports is interested
  Rule 3: if SCF is low and SSSQ is high, then sports is interested
  Rule 4: if SCF is medium and SSSQ is low, then sports is interested
  Rule 5: if SCF is medium and SSSQ is medium, then sports is highly interested
  Rule 6: if SCF is medium and SSSQ is high, then sports is highly interested
  Rule 7: if SCF is high and SSSQ is low, then sports is interested
  Rule 8: if SCF is high and SSSQ is medium, then sports is highly interested
  Rule 9: if SCF is high and SSSQ is high, then sports is highly interested

**Figure 6:**
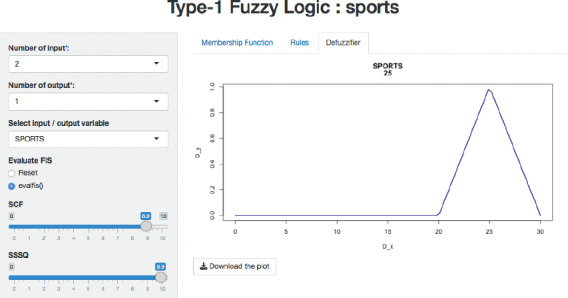
Users' interest prediction for the sports category.

**Figure 7:**
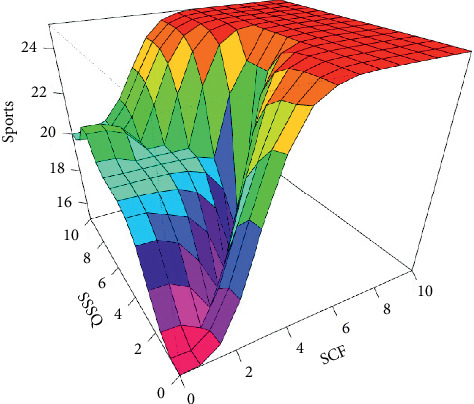
Surface graph SCF, SSSQ, and sports.

Based on the membership function values, all the rules in the rule-base of MFIS were executed. Then, the next step is modelling the fuzzy decision which combines all the antecedent parts and the consequent parts, thereby calculating the aggregation of output values. The resultant aggregating linguistic value was converted into a crisp value using the centroid defuzzification method and predicting the users' interest with respect to “SCF” and “SSSQ” as shown in Figure6.  Figure 7 illustrates the surface view of “SCF” and “SSSQ” and the interest prediction for the sports category reflecting the relationship between them. The same MFIS model was applied to predict users' interest in the following categories, namely, business, entertainment, technology, and politics with respect to its CF and SSQ.

### 3.3. Domain-Specific Popularity

The real-world events and news articles were spread and discussed by people effectively in an online social networking platform like Twitter and Facebook. These discussions were fruitful in the identification of ongoing viral and trendy news articles from social media. So, this proposed work is supportive for the determination of such viral news articles from harnessing social media feeds for the categories, namely, sports, business, entertainment, politics, and technology.

#### 3.3.1. Domain-Specific Viral News Identification from Facebook

News articles from various news portals can be posted or shared or liked by the Facebook customer and in their respective Facebook walls [37]. Thus, the news articles become viral in Facebook using share of articles and a like count. The viral news articles may be from different domains. So, it is necessary to identify such domain-specific viral news articles.

**Figure 8:**
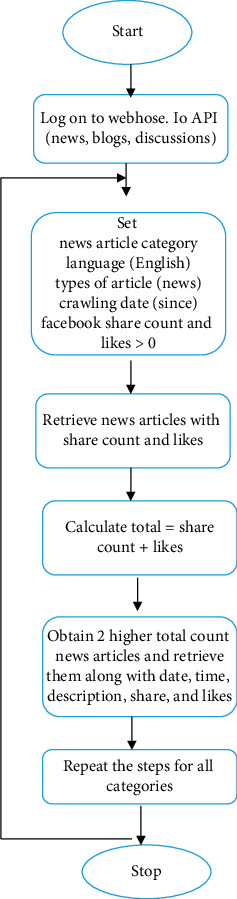
Retrieval of viral news articles from Facebook.

This work determines such viral domain-specific news articles from Facebook by using Webhose.io (social media monitoring API) [38]. It provides access to organized structured news articles shared or liked via monitoring Facebook media for multiple categories. Each category contains multiple subcategories. For instance, entertainment-relevant categories such as movies, lifestyle, and gaming are grouped under the entertainment category. The same thing is applicable for the other categories.  Figure 8 illustrates the retrieval of viral news articles from Facebook using webhose.io API. The likes and share counts keep on changing every day for the category of every news article. We have found share count and likes as limited for certain categories. So, the count was kept greater than zero. In this work, two topmost news articles were retrieved for the past three days up to the current minute for certain familiar categories.

Table 1 represents the details of performance of the domain-specific viral news articles from Facebook related to their categories. In order to evaluate this performance, two news articles were retrieved from each category. The retrieved news article categories pertaining to the respective categories were evaluated by using a news classifier.

#### 3.3.2. Domain-Specific Popular Hashtag and News Retrieval from Twitter

Generally, news articles were discussed by people in Twitter [39] through short messaging service known as tweets. Twitter users may group posts together by a topic or type under specific hashtags. The hashtags may become popular with the maximum number of tweets posted on those hashtags. Hashtags may come under different domains.

Moreover, several hashtags are available for each domain. It is necessary to identify such domain-specific related hashtags for each domain from Twitter. So, in this work, related hashtags for each domain were identified using the hashtag search engine tool.

In addition to this, Twitter crawling engine was built to retrieve tweet counts for each hashtag with respect to its domain during the period of last three days up to the present minute for the specific categories (as stated in Section 3.3.1) applying Twitter API. As a result, the popular hashtags were obtained with the help of the maximum number of tweets excluding retweets. Next, considering these popular hashtags, the two topmost tweets retrieved contain the maximum number of likes along with the corresponding news articles, Twitter account name, language, location, date, and tweeted time.  Table 2 represents the performance of popular domain-specific news articles retrieval of Twitter respective to its categories.

#### 3.3.3. Retrieval of Domain-Specific News Articles Rendered from News Feeds

The news articles were rendered from RSS feeds for all the categories from numerous news agencies like Times of India, British Broadcasting Corporation, and Cable News Network. The RSS feeds provide instant updated news article every time. The news articles rendered contain news snippet, description, date, time, and category.  Table 3 represents the details of performance of domain-specific news articles rendered from news feeds with respect to users' interested categories.

### 3.4. Recommendation of News Articles

MFIS was applied for the prediction of users' diversified interest in five categories. The users interested may belong to multiple categories. The following news articles recommended are as per users' interest categories:

1. Two popular news articles retrieved from Facebook along with title, description, category, date, time, share count, and likes
2. Two popular hashtag tweets (most likes) with corresponding news articles retrieved from Twitter along with date, time, and category
3. Latest time stamp news article along with title, description, published date, and time

## 4. Results and Discussion

The experiments were conducted on 25 users' IUP continuously for a period of 15 days to measure the performance of the proposed approach (MFIS). The main objective of this approach was to handle uncertainty from the IUP for predicting the users' interest and to classify them accurately in the following categories, namely, sports, business, entertainment, technology, and politics. The input attributes CF and SSQ for each domain were obtained from the IUP and fed as input for MFIS. Three linguistic variables were defined for both CF and SSQ using Gaussian membership function, and membership values have been represented in Tables 4 and 5, respectively. The linguistic variables were denoted as low 0–3, medium 3–7, and high 7–10. Similarly, three linguistic variables were defined for output attributes for specific categories using triangular membership function, and membership values have been represented in Table 6. The linguistic variables were denoted as not interested 0–10, interested 10–20, and highly interested 20–30.

Based on linguistic variables, 9 fuzzy rules were framed, and details are provided in Section 3.2. The rules were stored in the fuzzy associative memory table (FAM) as shown in Table 7.

Next, the defuzzification method, centroid, was applied, and it predicted users' interest precisely. For instance, the defuzzified crisp values are shown in Table 8 with respect to CF and SSQ.

**Figure 9:**
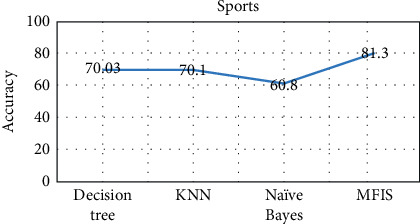
Users' interest prediction accuracy for the sports category.

**Figure 10:**
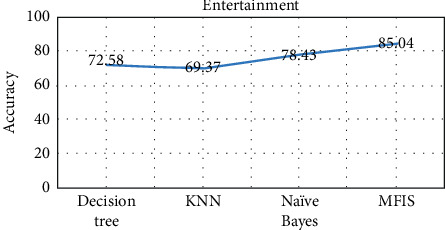
Users' interest prediction accuracy for the entertainment category.

**Figure 11:**
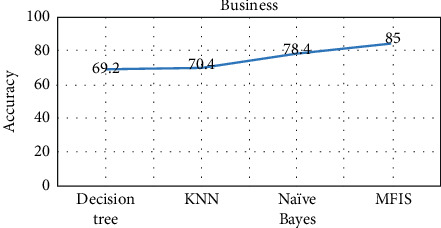
Users' interest prediction accuracy for the business category.

**Figure 12:**
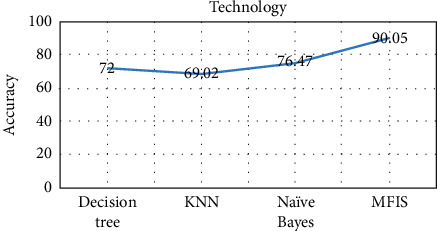
Users' interest prediction accuracy for the technology category.

**Figure 13:**
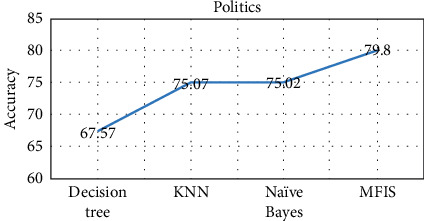
Users' interest prediction accuracy for the politics category.

**Figure 14:**
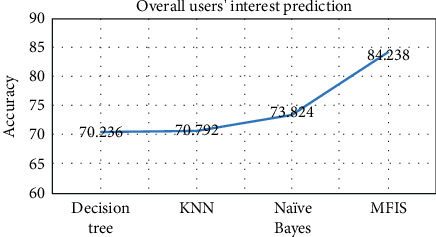
Accuracy of overall users' interest prediction for all categories.

The proposed approach was implemented in R programming using FuzzyR package, and prediction accuracy for all the domains was evaluated separately. The performance of the proposed approach MFIS was compared with the following classification techniques, namely, decision tree, KNN, random forest, and naïve Bayes, for predicting users' interested categories. In order to evaluate these classifiers' performance, the input dataset was first trained and then tested for the classification of users' interest categories for the following categories: sports, business, entertainment, technology, and politics. The performance of the proposed MFIS approach outperforms the users' interest prediction in all the categories when compared with the above classification techniques as shown in Figures 9–13. The overall users' interest prediction accuracy for all the categories of the proposed approach attained 84.238% as shown in Figure 14.

### 4.1. Recommendation Accuracy

We evaluated news articles' recommendation accuracy for 10 users randomly from 25 users. The viral and fresh news articles were retrieved for recommendation from Facebook, Twitter, and news feeds based on users' interest categories.

**Figure 15:**
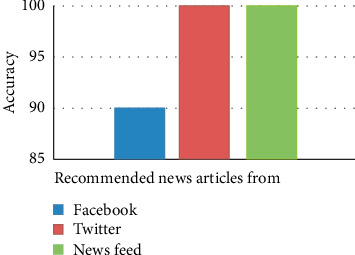
Overall news recommendation accuracy.

Moreover, it is necessary to identify the news article categories. We identified news article categories which were retrieved by using the news classifier. So, the overall performance of news recommendation was assessed on the basis of news articles retrieved from Twitter, Facebook, and news feed with respect to which users' interested categories are represented in Figure 15.

## 5. Conclusion and Future Enhancement

Traditional personalized news recommendations face two key challenges, namely, prediction of diversified users' interest and mining the social media for addressing popular news articles. This paper presents a MFIS approach for overcoming the challenges, for the prediction of users' interested categories, and identification of viral domain-specific news articles from harnessing Facebook and Twitter feeds, similarly, addressing the latest news articles from RSS feeds for specific categories. The experimental results have shown that the proposed approach (MFIS) attained overall 84.238% in the prediction of categories of users' interest. Then, the accuracy of overall news article recommendation from News Feed, Facebook, and Twitter attained 100%, 90%, 100%, respectively.

This work is limited to the prediction of users' interested categories only for a few domains. This work suggests extension to the prediction of users' interested categories for multiple domains for better recommendations.

## Figures and Tables

**Table 1:** Domain-specific viral news retrieval from Facebook.

S.no	Domain	Accuracy
1	Sports	100
2	Technology	100
3	Business	50
4	Entertainment	100
5	Politics	100

**Table 2:** Domain-specific popular news article retrieval from Twitter.

S.no	Domain	Accuracy
1	Sports	100
2	Technology	100
3	Business	100
4	Entertainment	100
5	Politics	100

**Table 3:** Domain-specific news articles rendered from news.

S.no	Domain	Accuracy
1	Sports	100
2	Technology	100
3	Business	100
4	Entertainment	100
5	Politics	100

**Table 4:** Membership values for CF.

CF	0	2.5	5	7.5	10
Low	1	0.25	0	0	0
Medium	0	0.25	1	0.25	0
High	0	0	0	0.25	1

**Table 5:** Membership values for SSQ.

SSQ	0	2.5	5	7.5	10
Low	1	0.25	0	0	0
Medium	0	0.25	1	0.25	0
High	0	0	0	0.25	1

**Table 6:** Membership values for the output category.

Output category	0	5	10	15	20	25	30
Not interested	0	1	0	0	0	0	0
Interested	0	0	0	1	0	0	0
Highly interested	0	0	0	0	0	1	0

**Table 7:** FAM table.

CF/SSQ	Low	Medium	High
Low	Not interested	Interested	Interested
Medium	Interested	Highly interested	Highly interested
High	Interested	Highly interested	Highly interested

**Table 8:** Defuzzified table.

CF	SSQ		Output
High (9) AND	High (10)	=>	Highly interested (25)
Low (2) AND	Low (2)	=>	Not interested (11.54)

## Data Availability

The dataset used in this study will not be provided by authors since users' privacy would be compromised.
